# Damage analysis of a perfect broadband absorber by a femtosecond laser

**DOI:** 10.1038/s41598-019-52432-x

**Published:** 2019-11-04

**Authors:** Ahasanul Haque, Monir Morshed, Ziyuan Li, Li Li, Kaushal Vora, Lei Xu, Lan Fu, Andrey Miroshnichenko, Haroldo T. Hattori

**Affiliations:** 1 The University of New South Wales, School of Engineering and Information Technology, Canberra, ACT 2612 Australia; 2 The Australian National University, Department of Electronic Materials Engineering, Research School of Physics and Engineering, Canberra, ACT 2601 Australia; 3 The Australian National University, Australian National Fabrication Facility, Canberra, ACT 2601 Australia

**Keywords:** Metamaterials, Metamaterials, Metamaterials, Metamaterials

## Abstract

Plasmonic metamaterial absorbers are particularly important in different applications such as photodetectors, microbolometers and solar cells. In this paper, we propose a tungsten boride (WB, a refractory ceramic) based broadband metamaterial absorber whose optical properties is numerically analyzed and experimentally characterized. We have also analyzed the damage characteristics of this absorber using a femtosecond laser and compared with an ordinary Au metamaterial absorber. We observe that WB has almost the double absorption bandwidth with absorption more than 90% over the spectral range of 950 to 1400 nm when compared with the Au counterpart. Furthermore, we show that Au metamaterial is damaged at the power of around 36.4 mW whereas WB metamaterial is not damaged at that power (WB has high Tammann temperature than Au)-however the atom of WB material was knocked off by the bombardment of a femtosecond laser.

## Introduction

Perfect absorbers are devices that absorb most of the incident electromagnetic wave and convert it into another form of energy such as heat. Since the demonstration of the original concept^1^, different types of absorbers have been proposed such as single-band^2^, dual-band^3^, multi-band^4^ or wideband absorbers. In fact, broadband absorbers are important in solar cell applications since solar cells need to absorb the broad solar spectrum and convert it into electricity. The advent of metamaterials brought new opportunities to the design of absorbers since they can tailor the electromagnetic properties of light^5–8^. In addition to absorbers, metamaterials have also been used to create many different devices such as perfect lenses^9^, sensors^2^ and solar cells^10–12^. The performance of metamaterial absorbers^13–18^ depends on their constituent materials and geometry. In recent years, different metals have been used in the metamaterial absorbers such as Au, Ag^19,20^ Cu and Ni^21^ because of their strong absorption at specific spectral ranges^22,23^. The problem with many of these absorbers is that they relied on strong but narrow plasmonic resonances, resulting in absorbers that could work on a limited range of wavelengths. These limitations pose a great constraint on the use of absorbers in solar cell applications, since they would need to absorb light in a wide range of wavlengths with small reflection^24–28^. Moreover, since solar panels are exposed to different types of conditions and temperatures, it is necessary that the chosen materials can operate at high temperatures^29^. It has been demonstrated that recently published devices presented very low efficiencies (0.8%^30^ and 3.2%^31^) mainly because of the overheating of the cells (up to 120 °C). Therefore, it is very important to pursue broadband absorbers which are stable under high power and temperature.

Different materials and approaches have been used to produce broadband and high temperature absorbers. For example, an ultra-broadband absorber based upon a refractory metal has been proposed by Gao *et al*.^32^. With adequate designs, absorbers operating at different wavelength regions have been produced: for example, Søndergaard *et al*.^33^ proposed an absorber operating at wavelengths between 450 and 850 nm with more than 96% absorption. Even nano-imprinting technique has been used to fabricate absorbers^34,35^. Typical metals such as gold and silver can produce broadband absorbers for photovoltaic applications if the design can work in a large bandwidth^36,37^ – however, these materials have low melting point around (1000 °C)^38,39^, meaning that the nano-structures of the absorbers might not operate at high powers^40^. On the other hand, some high temperature metals such as tungsten (W), molybdenum (Mo) and tantalum (Ta) might be suitable for operation at high temperatures and power^38,41^. However, they are not suitable for ideal absorbers because of their huge impedance mismatch (impedance of air and the device) in the visible to near infrared (NIR) region. We believe that Tungsten Boride (WB) can be a good choice of material for absorbers since it not only has plasmonic resonances but also possesses high melting point (2655 °C) in the visible to infrared region. In our case, we have used a *α*-WB-tetragonal tungsten monoboride (*α*-WB) which has the most stable crystalline structure of the material. Tungsten monoboride is a stable compound with phase-transition temperature of 2113 K^42^. It is also an inexpensive, super hard and lossy material^43,44^. By combining the plasmonic nature and large intrinsic loss of WB, we can design a broadband metamaterial absorber. Based on our knowledge so far, no one has used WB for broadband metamaterial absorbers and analyzed the damage threshold by a broadband femtosecond laser.

In this article, we design, fabricate and characterize a WB based metamaterial perfect broadband light absorber over a broad range of incident angles. The proposed metamaterial absorber with square structure shows polarization (patterns are symmetric) and angles insensitive (up to 60°) over the spectral range of 950–1400 nm. Most importantly, the fabricated WB metamaterial absorber shows extremly enhanced broadband absorption, demonstrating its potential in high power applications such as STPV.

## Design of the Perfect Absorbers

### General description of the devices

**Figure 1:**
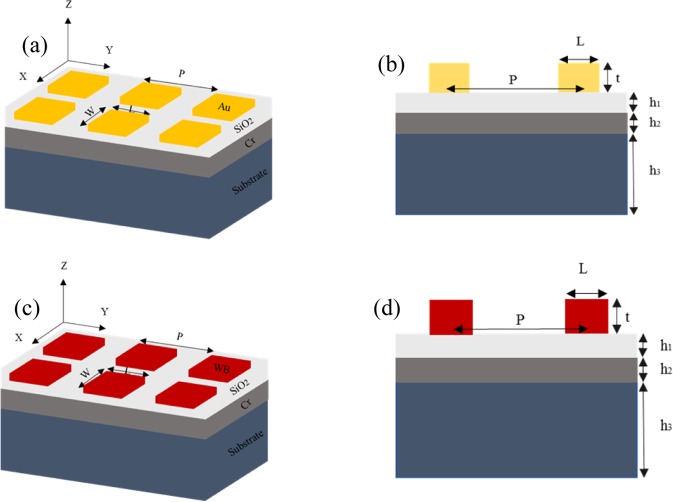
Geometry of the Au and WB metamaterial structures studied in this paper. The yellow regions are gold, and the light grey region is the dielectric and deep grey is chromium. W and L represent the width and length of each rectangular metallic structure along the x and y axis respectively, its thickness is represents by t, and *h*_1_ and *h*_2_ represent the thickness of dielectric spacer and bottom Cr layer respectively. P indicates the lattice constant. (**a**) 3D structure and (**b**) 2D cross sectional view of Au MMPA. (**c**) 3D structure and (**d**) 2D cross sectional view of WB MMPA.

The proposed metamaterials absorber was fabricated on a Si substrate consisting of three different material layers as shown in Fig. 1 metallic structures made of Au (yellow) or WB (Maroon) as a top layer, a *SiO*_2_ layer (light grey) of 140 nm in the middle, and the bottom Cr layer (deep grey) of 400 nm as a ground reflector for the absorber. The optimized geometrical parameters of the metallic structures are as follows: the length of each square L equals its width W as 322 and 400 nm, pitch of the device P is 900 and 1030 nm, and the thickness t is 100 and 200 nm for Au and WB, respectively. Chromium is used as a ground reflector for the absorber. Figure 1 shows schematic diagram of the designed metamaterial absorber devices with three dimensional (3D) and cross-sectional view (2D) in x-y plane of the patterned structures for Au and WB respectively. The proposed broadband metamaterials absorber consists of an array of square WB patterns periodically distributed in both x and y -directions with a period of P. The length and width of the square metallic particle along the x and y axis are consider to be equal (L = W). The patterned layer has been designed in such a way to fulfil the impedance-matching condition with the surrounding environment for zero reflection at the wavelength of interest. Also the symmetric patterns of square unit cell enables polarization independent absorption. The second layer is a dielectric spacer (*SiO*_2_) has been used to separate the two metallic layers. The *SiO*_2_ film is used to trap light efficiently (by changing effective refractive index). The *SiO*_2_ is a glass (100% transmission) and lossless dielectric and posses high melting point of 1600 °C. The bottom layer is a thick Cr layer of 400 nm for zero transmission and acts as a mirror. In order to find out the mechanism of the efficient light absorption behaviour of the WB metamaterial, we investigated the electric-field distribution in the structure. Figures S1 and S2 (see Supporting Information) show the electric field intensity profiles in the x-z plane and y-z plane at the square–dielectric spacer interface for the WB absorber at 1053 nm. We have also analyzed the absorption at different layer and observed that light have been efficiently absorbed by WB and thick bottom Cr layer as shown in the supplementary information of Fig. S3.

The optical properties of WB used during the simulation were experimentally measured by an ellipsometer under elliptically polarized white light in the range of 190 to 1680 nm. We have used (Glass)-palik^14^ model in order to define the properties of *SiO*_2_. It is noted that all materials used in this paper are considered to be nonmagnetic. The metamaterials absorber has been designed with Finite-Difference Time-Domain (FDTD) Lumerical software. The proposed absorber has been designed with operating wavelength at near 1053 nm and optimized parameters for WB are taken as L = W = 400 nm, t = 200 nm, *h*_1_ = 140 nm, *h*_2_ = 400 nm, and period = 1030 nm. For the optimization of the proposed structure thickness, initially we have kept the length and width constant and changed the thickness. Similar method was also used to optimize other parameters such as length, width and period. Variation of the absorption for different period and length (or width) and period has been added in the Figs S4 and S5 in the Supporting Information, respectively. The incident light can be either reflected (back to the source media), transmitted or absorbed by the materials. The absorption can be calculated by the following formula: A = 1-R-T; where A, R and T represents absorption (A), reflection (R) and Transmission (T) respectively. It means that to obtain absorption near unity, both the reflectance and transmittance need to be reduced to near zero. The reflected energy would only be zero when the impedance of the plasmonic structure are perfectly matched to the free space wavelength and at the same time the optical resonance would be stimulated. Such resonances may efficiently trap light energy and provide enough time to dissipate it by dielectric or ohmic loss when the structures are thick enough, which means that light is consumed and therefore turned into dissipated heats. Recent attention has also been drawn to the generation of heat in plasmonic structure caused by light absorption^25,45,46^.

**Figure 2:**
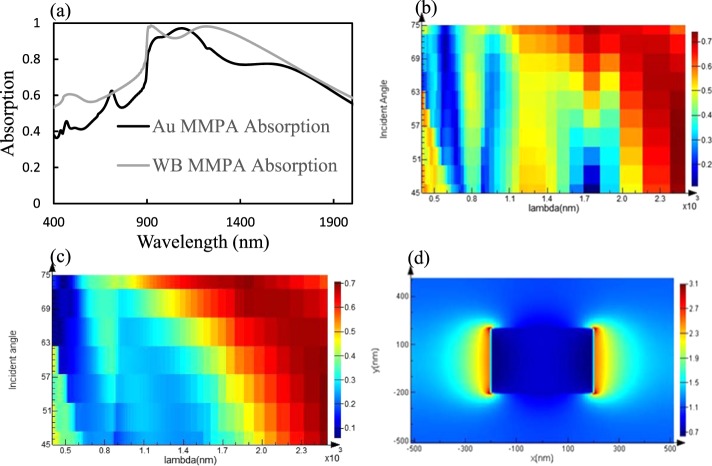
Au and WB metamaterial absorbers. (**a**) Simulation results of the Au and WB perfect absorber absorption extracted from FDTD simulations of a periodic structure with a period of 900 nm and 1030 nm respectively. (**b**) 2D plot of reflectivity with angle vs wavelength for Au MMPA (**c**) 2D plot of reflectivity with angle vs wavelength for WB MMPA. (**d**) Electric field intensity inside the WB structure at 1053 nm.

Figure 2 indicates theoretical analysis for Au and WB metamaterials perfect absorbers. The metamaterial absorber proposed in Fig. 2(a) was theoretically design using a commercial FDTD Lumerical software. In Fig. 2(a), the simulated absorption of the WB metamaterial above 91% over the whole visible range of 950–1400 nm, and for most wavelengths the absorption is larger than 90%. Approximately unity absorption is attained at about 1053 nm, and the overall absorption is around 90% in this spectral range. Figure 2(a) shows that WB absorption bandwidth is larger than the Au (almost double) and WB absorbed above 90% light over the spectral region from 950 nm to 1400 nm. Placing periodic structure metallic resonators along the horizontal direction allows broadband perfect absorption due to the mixing of multiple resonances. The broad bandwidth also may be due to the wave diffraction at diagonal corners which can be further enhanced by multi-layer overlapping and regulation of period length between adjacent unit cells. On the other hand, Au absorbed light above 90% in over the spectral range of 1000 nm to 1200 nm. Figure 2(b) shows 2D reflectivity plot with incident angle vs wavelength which indicates reflection changes with incident angle. Figure 2(c) shows the 2D reflectivity plot with variable incident angle at different wavelength and has been observed that WB has wider bandwidth as compared to Au. Figure 2(d) confirms the field profile of WB at 1053 nm and indicates perfect absorption and localized plasmonic excitation near the edges of the structure.

## Sample Fabrication

**Figure 3:**
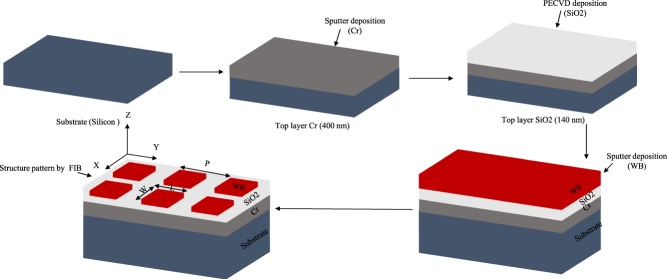
Fabrication Process flow diagram (WB structure length and width 400 nm and thickness of 200 nm with period of 1030 nm).

Figure 3 shows the fabrication processes flow of the metamaterials absorber devices. To fabricate the WB metamaterial absorber, an RF Magnetron sputter system was used to deposit 400 nm Cr on a silicon substrate with a deposition rate of 2.58 nm/min. Then a *SiO*_2_ layer of 140 nm was deposited by a plasma enhanced chemical vapor deposition (PECVD) system with a deposition rate of 48 nm/min. The thickness of each material were measured by an ellipsometer except Cr. After that, 5 nm Cr as an adhesion layer and 200 nm WB was deposited on top (deposition rate of WB was 4.8 nm/min) by the sputter and measured thickness by surface profiler. Finally, we patterned square structure on the WB layer by focused ion beam (FIB) milling. On the other hand, 100 nm Au metallic structures were obtained by electron-beam deposition followed by electron-beam lithography (EBL) and lift-off process as an experimental reference.

## Experimental Results and Discussion

**Figure 4:**
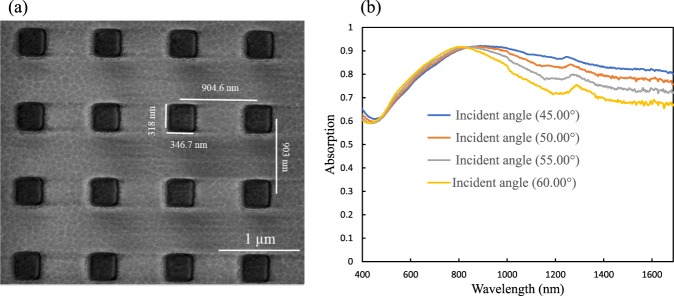
Gold metamaterial absorber. (**a**) Fabricated SEM image of Au pattern. (**b**) Experimentally measured absorption at different incident angle.

The nature of the proposed metamaterial absorber is similar to the infrared absorber demonstrated in^14^: both are attributed to the excitation of the localized electromagnetic resonant mode. The mechanism can be explained as the localized electromagnetic fields which are strongly enhanced at resonances. The incident light is trapped efficiently at each cell’s edge. The proposed device is independent of polarization of normally incident light due to the symmetrical pattern (the length and width of the WB particle are set to be equal in x and y axis)^14^. Moreover, the absorption is also strong for inclined incident angle for such type of absorber. To experimental demonstration of our method, a variable angle spectroscopic ellipsometer (V-VASE) was employed for reflection measurement with optional focusing probes that focus the beam to a spot size diameter of around 300 µm. Therefore, we prepared the metallic particle array with a size of about 400 × 400 μ*m*^2^ to make sure the whole measured area was covered. Figure 4(b) shows the experimentally measured absorption as a function of wavelength and different incidence angle for fabricated Au metamaterials as shown in Fig. 4(a). Strong absorption still valid for large incident angle for instant when the incident angle is 60 degree the absorption remains around 91%. This is because of the fact that the orientation of the magnetic field changes with the incident angle. However, for s-polarization the absorption is changing because the orientation of the magnetic field does not change with the incident angle.

Figure 4(b) shows that the measured absorption which has better agreement between simulation (Fig. 2(a)) and experiment (Fig. 4(b)) except for certain amount of absorption which may be due to the fabrication error. Although we have numerically analyzed with sharp square array of gold, the corner of the fabricated Au structures are round with about 4 nm radius and the length and width were found to be 346.7 nm and 318 nm respectively due to the fabrication limitation.

**Figure 5:**
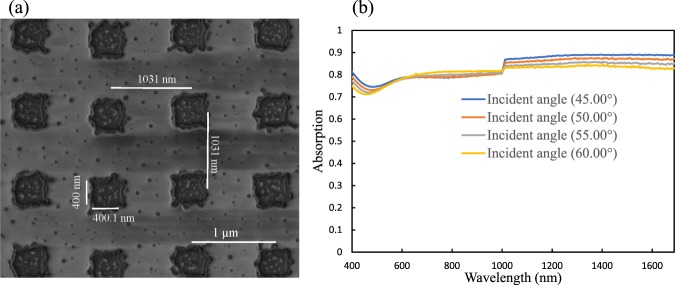
Broadband WB metamaterial absorber (**a**) Fabricated SEM images of WB pattern (**b**) Experimentally measured absorption.

Figure 5 displays the fabricated WB metamaterial absorber. The measured dimensions are near to the simulated design except some unavoidable error around ±5 nm for length, width and thickness. Moreover, the fabricated patterns is not sharp as the designed structure. The radious of the round at the corner was approximately 4 nm because of fabrication error. Due to the hardness of the WB materials it is hard to do FIB milling even at higher current. As a result, the square shape is not exactly square but with certain roughness. Figures 2(a) and 5(b) has better agreement between simulation results and experimental results with small discrepancy which may be due to fabrication error. There is poor different between the measured and simulated absorption spctra due to the minor variation in the dimensions as well as slightly changes in the optical properties of the fabricated structure.

**Figure 6:**
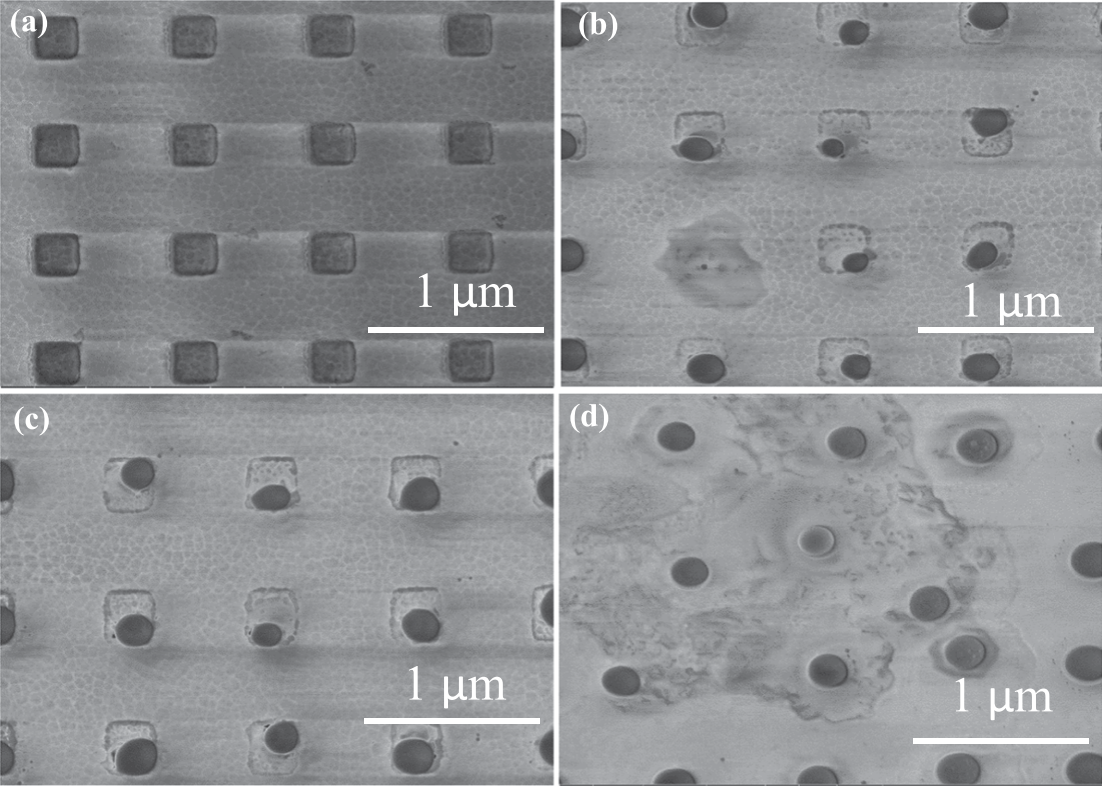
SEM images of the studied gold metamaterial absorber (**a**) without laser exposure and (**b**) under 36.4 mW clearly indicating melting of absorber (**c**) 46.42 mW (**d**) 52.14 mW.

Figure 6 shows the Au metamaterials perfect absorber for different applied power from a femtosecond laser experiement (laser with pulse width of 200 fs, 80 MHz repetition rate and working wavelength is 1100 nm). Figure 6(a) shows fabricated structure pattern with no exposure to laser. When the applied power was increased to 36.4 mW, the Au pattern started to melt and gradually was converted to circular nanoparticle with further increase in power. These structural change can be observed from Fig. 6(b–c).

**Figure 7:**
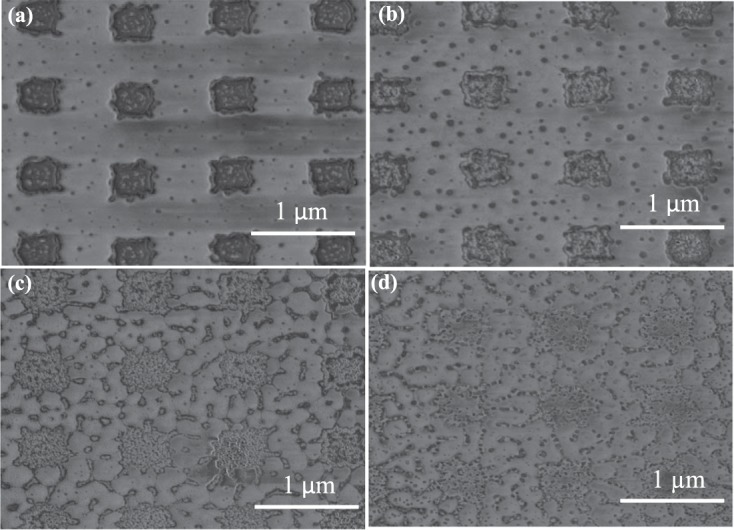
SEM images of the studied WB metamaterial absorber (**a**) without laser exposure and (**b**) under 25.43 mW (**c**) 28.57 mW (**d**) 39.29 mW.

**Figure 8:**
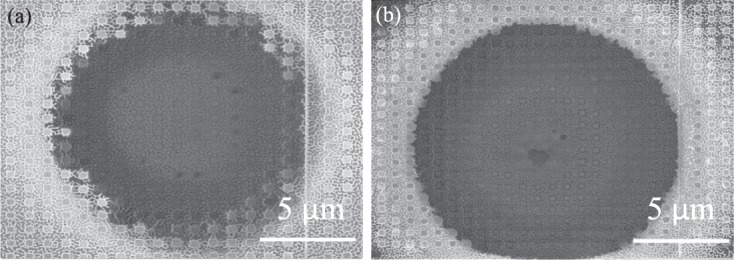
SEM images of the studied WB metamaterial absorber (**a**) 46 mW (**b**) under 85 mW.

In contrast, Fig. 7 illustrate the WB patterns for different applied power. Figure 7(a) shows WB pattern without exposure. However, when we applied 25.4 mW it shows some changes in the structure but patterns are still intact. After increasing a certain level of power, we observed that WB pattern displaced from its original location. For further investigation we applied around 46 mW and 85 mW as shown Fig. 8(a,b) respectively. From the Fig. 8, it can be observed that at the center of the laser beam WB material is removed but at the periphery structure still remain good. This can be due to the fact that the applied laser beam has Gaussian shape and hence the peak power is at the center point. The measured spot diameter is around 10 Âµm and the peak intensity has been calculated by Gasussian beam power and intensity equation which is given below:

1 $$I(r,z)={I}_{0}\mathrm{(1}-{e}^{\frac{-\mathrm{(2}{r}^{2})}{\omega {(z)}^{2}}})$$

where, I is the intensity and r indicates radius at z position. The beam transmitted power is given by *I*_0_. The maximum amount of materials has been damaged at the central due to high intensity for Au, but WB metamaterials does not melt. WB does not melt as it is very hard material and has melting point of 2665 degree Celsius. However, from the figure it is evident that, WB material is removed which may be due to the melting of Cr thin layer. WB metamaterial is not damaged however- the atom of WB material is knocked off by the bombardment of femtosecond laser due to weak adhesive layer. On the other hand at the center, the WB material swipe out due to the weak adhesion layer as we have used a thin layer of Cr (5 nm) for both Au and WB metamaterials absorber. These indicate WB materials do not melt but displace some of the pattern due to weak adhesion layer between WB and *SiO*_2_. The intensity for certain point (distance from the central of the beam is z) for Au and WB is calculated as 1.0480 mW/m^2^. From the Fig. S6 in the supporting information it can be observed that Au pattern has been damaged. Although WB damaged at a lower power, we suspect it was because of Cr layer which is necessary as an adhesion layer. It is counter intuitive because WB has higher melting point. Another reason would be WB has broadband absorption and absorbed higher power from femtosecond laser. However, a portion of WB material was knocked off by the bombardment of femtosecond laser due to weak adhesion layer (at the central point of the beam has high intensity that’s why 5 nm adhesion Cr layer melt).

## Conclusion

In conclusion, we have designed and characterized a broadband metamaterial absorber based upon tungsten boride (WB), which has a measured absorption of 90% in the wavelength range between 950 and 1400 nm. The broad absorption spectrum is the result of the coupling of different resonances, with the interplay of WB material properties allied with the metamaterial geometry. In addition we have analyzed damage of the metamaterial by a femtosecond laser’s high power exposure – it is damaged at an average power of 28 mW. The wideband WB absorber has the potential of being used in solar cell applications and in other application which require absorption of light over a wide range of frequencies.

## Methods

The numerical analyses of our proposed metamaterials perfect absorber are conducted by using commercial three-dimensional finite difference time-domain (FDTD) based Lumerical software with periodic boundary conditions. The whole structure is surrounded by air and the incident wave is assumed to be a plane wave. The spot size diameter of the source is much larger than the computational area of the structure. The mesh size is chosen as Δx = Δy = 20 nm and Δz = 5 nm. Figure 3 shows the fabrication processes flow of the metamaterials absorber devices. To fabricate the WB metamaterial absorber, an RF Magnetron sputter system (Sputter Coater system-AJA) was used to deposit 400 nm Cr on a silicon substrate with a deposition rate of 2.58 nm/min. Then a *SiO*_2_ layer of 140 nm was deposited by a plasma enhanced chemical vapor deposition (PECVD) system with a deposition rate of 48 nm/min. The thickness of each material were measured by an ellipsometer except Cr. After that, 5 nm Cr as an adhesion layer and 200 nm WB was deposited on top (deposition rate of WB was 4.8 nm/min) by the sputter and measured thickness by surface profiler. Finally, we patterned square structure on the WB layer by focused ion beam (FEI Helios NanoLab 600 dual beam focused ion beam (FIB) system) milling. On the other hand, 100 nm Au metallic structures were obtained by electron-beam deposition followed by electron-beam lithography (Electron Beam Lithography (EBL-Raith 150) system) and lift-off process as an experimental reference. To experimentally demonstration of our method, a variable angle spectroscopic ellipsometer (V-VASE) was employed for reflection measurement with optional focusing probes that focus the beam to a spot size diameter of around 300 Âµm. Therefore, we prepared the metallic particle array with a size of about 400 × 400 µ*m*^2^ to make sure the whole measured area was covered. Then the damage threshold have been measured by a femtosecond laser experiement (laser with pulse width of 200 fs, 80 MHz repetition rate and working wavelength is 1100 nm). We have applied different power level to find out damage threshold and each time we have checked SEM images to ensure the damages.

## Supplementary information

Supplementary Info
