# Supplementary material for: Damage analysis of a perfect broadband absorber by a femtosecond laser
Source: Sci Rep. 2019 Nov 4;9:15880. doi: 10.1038/s41598-019-52432-x (PMC6828741; doi:10.1038/s41598-019-52432-x)
Supplement: Supplementary file 1 — Supplementary Info [file 41598_2019_52432_MOESM1_ESM.docx]

**Supporting Information**

Damage analysis of a perfect broadband absorber by a femtosecond laser.

Ahasanul Haque1*, Monir Morshed, Ziyuan Li, Li Li, Kaushal Vora, Lei Xu, Lan Fu, Andrey Miroshnichenko and Haroldo T. Hattori


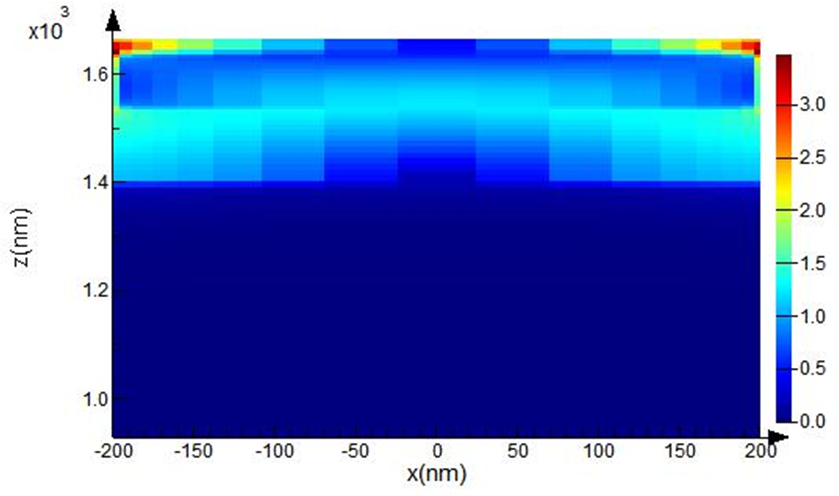


Figure S1. Electric field profile in the x-z plane for WB pattern at 1053 nm.


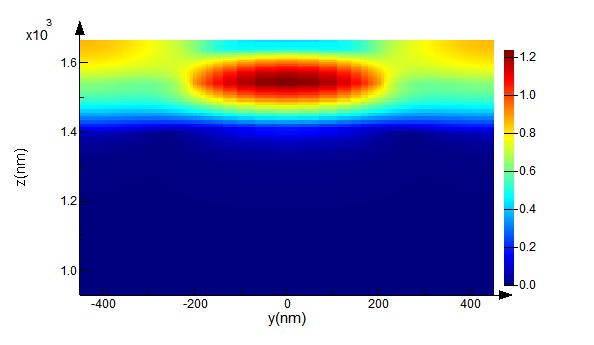


Figure S2. Electric field profile in the y-z plane for WB pattern at 1053 nm.

Figure S3. Absorption contribution from the top WB structure and the back layer for the Cr absorber.

Figure S4: Simulated absorption spectrum at different period.

Figure S5: Simulated absorption spectrum at different length (or width).


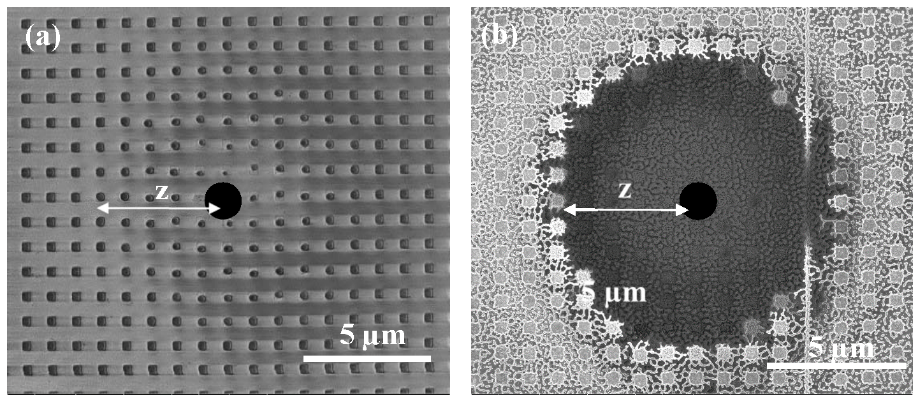


Figure S6: SEM images of the studied (a) Au and (b) WB metamaterial absorber at the power of 36.4 mW and 39.3 mW respectively.
